# Diseases of the Gums

**Published:** 1893-02

**Authors:** John L. Gish

**Affiliations:** Jackson, Mich.


### Diseases of the Gums.

Read in the Section of Oral and Dental Surgery, at the Forty-third Annual Meeting of the American Medical Association, held at Detroit, Mich., June, 1892.

BY JOHN L. GISH, M.D., D.D.S.,
of Jackson, Mich.

It is not my purpose to try and cover the whole field of pathological conditions that would naturally come under the above heading, but simply take up such parts as are more common in our general work

So far as the anatomy, histology and normal conditions of the gums are concerned, I will pass over that division and not consume time by repeating that which we must know in order to fully appreciate any abnormal conditions or changes that may take place in the gums.

When you deal with pathological conditions, let them exist wherever they may, in our body, we at once encounter the problems of growth and development, nutrition and assimilation. So here, when we find diseased conditions of the gums, we know that the laws that govern the process of growth and development, and the functions of nutrition and assimilation, have been changed to a greater or less extent, with the corresponding resultant of active or passive congestion, hypertrophied or atrophied conditions or increased cell growth of the tissues involved.

When we take up active or passive congestions or the acute and chronic inflammations of the gums we consider the type of cases that come to our notice more often, and cause more trouble to our patients than is made manifest by the hypertrophied, atrophied and cell growth conditions combined. Under the section of acute inflammation of the gums we have to contend with the acute or inflammatory œdema, marked by its rapidity of development, severe pain, tumefaction and great tension of the tissues, due to some chemical or traumatic lesion, and closely connected to this pathological condition, we do encounter gangrene of the mouth (or noma), which is less rapid in its development and attended with a less degree of pain, but far more destructive in its results, and much more obstinate in yielding to treatment. Under this division we may also include the acute catarrhal, herpetic and croupous inflammations.

Under division second, or passive congestions, where the circulation is slowed in its course, affording an opportunity for the red blood corpuscles to accumulate in the arteries, and the white blood corpuscles to find their way into the veins, whose anatomical structure, viz: their thin walls afford an easy escape of the white blood corpuscles, by their amœboid movements, into the surrounding structures, and at the same time with the passage of the white blood corpuscles or leucocytes, there is also the transudation of the liquor sanguinis, forming the serous effusion, which, together with the leucocytes, dividing and multiplying, excite the tissues into cell-proliferation. This in turn causes a greater nutritive activity with the formation of a cell-growth, which is very unstable in its composition and soon passes into retrogression, or the breaking down stage, with the formation of pus, which dissolves alike the alveolar process, the dental organs and the soft structure, thus completely destroying the involved tissues and ending a cycle of changes, only again to be renewed and cause further destruction of the parts by continuity and contiguity, as is done in chronic catarrhal inflammations, phlegmonous and suppurative inflammations or abscesses, and fistulæ of the gums.

Under division three we will include hypertrophied and atrophied conditions, and under division four such cell growths as the fibrous, papillary, and vascular tumors and epitheliomata of the gums.

Division four, including as it does, the variety of cases that come to our notice the least, have pathological conditions in common, varying only in the elements involved, and I cannot do as well in giving their pathology, as I can to repeat the words ot Wedl, who regards papillomata of the gums as essentially localized hypertrophies of the papillary portion; generally associated with caries and located upon the facial surface of the gum; their diminutive conical elevations form dome-shaped enlargements and present an analogy with acuminate condylomata.

Fibromata of the gum occur in the submucous connective tissue in the form of projecting, superficially smooth, compact tubercles which slope away towards the surrounding tissues; they are by no means of rare occurrence, especially those of smaller dimensions, and are composed of firm bundles of connective tissue with numerous interlacements and imbedded cells. Linhart extirpated a fibroma of the submucous connective tissue nearly as large as a hen's egg.

Angiomata (vascular tumors) are of rare occurrence and some give rise to hemorrhage, which endangers life. Salter reported a new-formation of this kind which was composed of numerous convoluted blood-vessels and connective tissue. The surface of the tumor, which was lobulated, located upon the neck of a tooth and as large as the berry of the common winter-cherry, was covered with papillæ like those of the gum, and with epithelium. The latter condition serves to indicate that the new formation was seated in the submucous connective tissue of the gum, and not, as alleged, in the dental periosteum. Epithelial cancer sometimes occurs primarily in the gum and knotty elevations are developed, which increase in number and extent, and spread to the submucous connective tissue, dental periosteum, alveolus and alveolar process. The epithelium undergoes notable proliferation and imparts a brighter color to the knots; it also dips down to a considerable depth into the substance, where the familiar rosette-shaped, nest-like groups of flattened, often distinctly ribbed cells are met with. Sometimes the papillæ of the gum also grow considerably in length and breadth, and the cancer acquires the appearance of a succulent wart which finally ulcerates. Schuh observed the origin of the epithelial cancer upon the buccal surface, usually in the vicinity of the last molars. Here also, he says, it maintains for a time the character of the broad cancer; that is, of a form of cancer which increases only in its extent of surface, not in depth, remains superficial, therefore resembling a granulating surface, and slowly destroys the organic parts.

The hypertrophied and atrophied conditions, or division three of this paper, are more often met with than the conditions under division four, and of the two types of conditions under division three, the hypertrophied is far more common.

Atrophy, as the name implies, is a progressive and morbid diminution of the bulk of tissues of which the gum is composed. Atrophy is generally symptomatic, but it may be due to a local cause. Now, inasmuch as atrophy from a pathological standpoint is just the reverse of hypertrophy, it will not be necessary to review its general pathology in order to state the changes that take place, but the general pathology of hypertrophy of the gums as given by Wedl, will serve the double purpose; hence, hypertrophy of the gums occurs frequently among the sequelæ of a chronic inflammatory condition of the root membrane following caries of the teeth, and is induced, also, by the irritation of the gums produced by the products of the degeneration of the hard tissues of the teeth.

The sharp edges of the stumps, too, which remain after the destruction of the coronal portions and necks of the teeth, become the sources of irritations.

The hypertrophy occurs both in the papillary portion and beneath the latter, in the corium of the mucous membrane. When it occurs in the former, it is most conspicuous upon the facial surface of the gums, and is confined to the region of one tooth or a few teeth, or acquires more extended limits in cases of caries involving the crowns or roots of several teeth.

The epithelium, also, participates in the hypertrophy which affects the papillary portion.

The secretion of mucous usually is increased in connection with papillary hypertrophy, and partial exfoliations of the epithelium occur, leaving superficial erosions. Bleeding readily occurs from the painless swellings, even from slight mechanical irritations. Sometimes the papillary portion is not involved and the proliferation is confined to the corium, occasioning a swelling of the gum which has a smooth exterior. In these cases, which are more protracted, the cellular infiltrations rise to a tendinous connective tissue; in other words, the hypertrophic gum becomes sclerosed, and, consequently, it presents an increased power of resistance, and a diminished succulency. The gradually obliterated papillary portion is covered by a common, thick, superficially smooth layer of epithelium. Notwithstanding the increased consistence of the corium, cellular-infiltrates are met with in the form of long, chain-like rows or clusters, of ovoid cells, which are enclosed with a capsule of fibrous tissue. Since the periosteum, also, beneath the indurated gum is thickened, it is by no means strange that cross sections of the latter sometimes present slender trabeculæ in the process of growth, together with young bone-corpuscles," while the above has given us the general pathology of hypertrophic conditions of the gums, there is yet another subdivision of this type of cases that does come to our notice quite frequently, and I mean the hypertrophied conditions of the tissues that oftentimes surrounds, or accompanies the eruptions of the wisdom tooth. It is true that in this particular type of cases we have a greater degree of pain, more swelling, and inflammatory action extending to the surrounding tissues, than is the case in true hypertrophy of the gums, but the difference in the exciting cause will explain the difference in symptomatic results.

In true hypertrophy, usually the result of some chronic inflammation of a root membrane, or the irritation of the gums produced by the products of the degeneration of the hard tissues of the teeth, we have the train of symptoms or conditions as above given; but in this particular type of cases caused by undue pressure on the surrounding tissues, and still further augmented by the close relations of the parts, and the mechanical action of mastication we account for the difference in subjective symptoms.

In chronic catarrhal inflammation, included under division second, or passive congestions, we find the mucous membrane thickened and of a rose color with white spots which appear and disappear with the exfoliation of the epithelium. The patient complains of unpleasant sensations, such as burning, itching and tickling, and as a result of this congestion there is a gray, mucilaginous, and at times a puriform secretion poured out upon the surface of the gums. Of course, the degree of this inflammation is modified to a greater or less extent, according to the time that the lesion is allowed to run, and as to whether the patient is suffering from other systemic troubles, or from catarrhal inflammations of surrounding membranes.

Phelgmonous inflammation is somewhat sudden in its onset, but is attended with but little or no fever. The congestion is confined to both the corium and submucous connective tissue, with ulcerations occurring in small, shallow, yellowish spots or in the form of one large phagedenic ulcer. If this ulceration is allowed to continue, we will have necrosis of the osseous substance beneath. Recovery is very slow, and the surrounding membrane is spongy, from infiltration of the liquor sanguinis, very sensitive and bleeds easily. This type of cases is common and is found in the well nourished, as well as in those who have systemic complications.

Suppurative inflammation is where the lesion has been such as to cause the passive congestion to result in the formation of pus. This pus may find its way to an external opening or it may undergo resorption. If the exciting cause has been mechanical, such as compression, contusion or laceration, the recovery is speedy with suppuration, but if on the other hand, the exciting cause is a diseased root membrane of a chronic character, and the irritant becomes extended to the gum, a fistula is formed, which will continue to discharge its secretion until the parts are made healthy within.

Again referring to division one, under which we consider inflammatory œdema, acute catarrhal, herpetic and croupous inflammations, there is in common the rapidity of onset, severe pain, tumefaction and sponginess of gums, attended with the unpleasant sensation of itching and burning. In addition to this, the herpetic inflammation is characterized by the formation of vesicles, which rupture and remain a superficial erosion, healing without cicatrization. Closely allied to the herpetic condition is the inflammatory affection of the gums, developed during the course of an acute exanthemata, such as scarlet fever, measles, etc.

The croupous inflammation not only has the subjective symptoms that attend all active congestions, but it is characterized by the grayish structureless membraniform exudation, which may be very easily detached. This exudation takes place very rapidly, and as rapidly undergoes degeneration with the production of an offensive, sanious mass, which dips down between the gum and the tooth, thus exciting the root membrane into an active congestion with the corresponding loosening of the tooth. If the action is not checked, the alveolar process is destroyed and the teeth are lost.

In considering the treatment of the above pathological conditions, it must be governed according to the nature and type of the case in hand. But to generalize I will say that in treating division first, or active congestions, with the exception of those cases due to acute exanthemata, I have found in galvanic and faradic electricity my most valuable agent, and it is so valuable, from the fact that by the proper manipulation of this subtle agent we can correct circulatory disturbances, promote absorption of the transudated fluids, and cause the secretions and excretions to be changed into their normal condition.

Take, for instance, a case of noma, destructive in its results, and obstinate to cure, here by an application of electro-chemical cauterization from the positive pole, we can arrest the superficial destruction of tissue, and produce a healthy restoration of the parts beneath.

In treating the passive congestions I can apply electricity with benefit only to the chronic catarrhal inflammations, the other types of cases of the second division are treated according to the principles of good surgery.

In dealing with hypertrophies, fibrous tumors and epitheliomata the electro-cautery has been my best and most successful remedy. In order to cope with the papillary and vascular tumors, the galvanic current has been my most potent agent.

